# Effect of Doping Heteroatoms on the Optical Behaviors and Radical Scavenging Properties of Carbon Nanodots

**DOI:** 10.1021/acs.jpcc.3c00953

**Published:** 2023-04-05

**Authors:** Mahsa Azami, Jianjun Wei, Mehrab Valizadehderakhshan, Anitha Jayapalan, Olubunmi O Ayodele, Kyle Nowlin

**Affiliations:** † Department of Nanoscience, Joint School of Nanoscience and Nanoengineering (JSNN), University of North Carolina at Greensboro, Greensboro, North Carolina 27401, United States; ‡ Joint School of Nanoscience and Nanoengineering (JSNN), North Carolina Agricultural and Technical State University, Greensboro, North Carolina 27401, United States

## Abstract

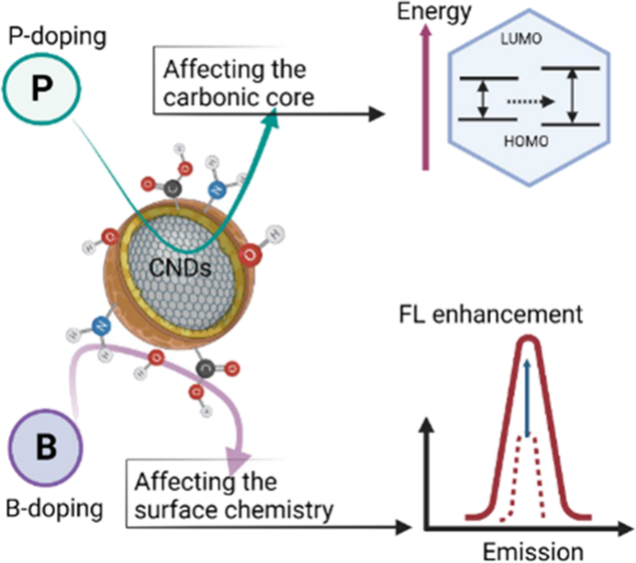

Heteroatom doping is regarded as a promising method for controlling the optoelectronic properties of carbon nanodots (CNDs), notably their fluorescence and antioxidation activities. In this study, phosphorous (P) and boron (B) are doped at different quantities in the CNDs’ structures to investigate their effects on the optical and antioxidation properties. Both the dopants can enhance light absorption and fluorescence, yet via different approaches. After doping, the UV–vis absorption of high P%-CNDs demonstrated a slight blue shift (348–345 nm), while the high B%-CNDs showed a minor red shift (348–351 nm), respectively. The fluorescence emission wavelength of doped CNDs changes marginally while the intensity increases significantly. Structural and composition characterizations show elevated levels of C=O on the surface of high P%-CND compared to low P%-CNDs. In B-doped CNDs, more NO_3_^–^ functional groups and O–C=O bonds and fewer C–C bonds form at the surface of high B%-CNDs compared to the low B%-CNDs. A radical scavenging study using 2,2-diphenyl-1-picrylhydrazyl (DPPH) was carried out for all CNDs. It was found that the high B%-CNDs exhibited the highest scavenging capacity. The effects of the atomic properties of dopants and the resulting structures of CNDs, including atomic radius, electronegativity, and bond lengths with carbon, on the optoelectronic property and antioxidative reactions of CNDs are comprehensively discussed. It suggests that the effect of P-doping has a major impact on the carbogenic core structure of the CNDs, while the B-doping mainly impacts the surface functionalities.

## Introduction

Carbon nanodots (CNDs) are zero-dimensional carbon nanoparticles typically less than 10 nm in size.^1^ CNDs have piqued the interest of researchers owing to their tunable optoelectronic features, especially high intrinsic fluorescence, along with their biosafety, making these moieties promising candidates for a wide range of applications, including biomedical,^2,3^ electrocatalysis, and energy storage.^4^ The optical behavior of CNDs is affected by a combination of variables, i.e., precursors, synthesis procedures,^5^ final particle size,^6^ surface functional groups, and doping with various heteroatoms.^7^ Among these, surface chemistry appears to have a significant impact on the optical properties of CNDs. The fluorescence of CNDs stems from photon absorption in their carbonaceous core, which then incorporates with the charge recombination at the surface.^8^ Although the role of the amorphous carbonaceous core of CNDs in their optical performance is not negligible, it has been demonstrated that surface manipulation of CNDs may induce more significant changes in the emissive patterns, thereby promoting the fluorescence features.^7^ The effect of surrounding functional groups also seems to be complicated. Forming dipole moments resulting from functional groups on the CNDs’ surface could affect the properties of CNDs by not only the intrinsic dipole moment of functional groups but also the induced dipole formed between the functional groups and the carbogenic core edges.^9^ Although it is difficult to predict, it is generally believed that functionalization—especially with groups containing oxygen and nitrogen—can affect the π–π* and n−π* electronic transitions more than the others outlined in CNDs (e.g., σ–σ*, σ–π*, and n−σ*).^10−12^ As one of the optoelectronic features, the antioxidation properties of CNDs can also be altered by manipulating the functional groups. CNDs are usually known as n-type (electron donor) moieties since they may donate electrons to other materials. This feature of CNDs, which is primarily the result of surface functional groups, is the basis of their antioxidation characteristics, allowing them to function as radical scavengers. For example, Ji et al. studied the individual effect of COOH and NH_2_ surface functional groups on the radical scavenging potential of CNDs, through which the direct or indirect hydrogen atom transfer could be the possible mechanism engaging these functional groups in the radical scavenging reactions.^13^

Doping CNDs with heteroatom elements is one of the effective ways to modify their optical properties since it can create changed electronic states in their carbonic framework structures and influence their surface chemistry as well.^14^ Doping can alter the highest occupied molecular orbital (HOMO) and lowest unoccupied molecular orbital (LUMO) levels. Monitoring variations of the band-gap energy levels driven by doping allows for analyzing the function of these dopants on the structure and, as a result, modifying the optical characteristics of CNDs.^15^

Nitrogen (N), phosphorus (P), boron (B), and sulfur (S) are the closest neighbors to carbon (C) in the periodic table, which have been applied widely for doping different types of CNDs, converting them to practical tools for a broader range of applications.^16−19^ The antioxidation potency of CNDs is also influenced by the dopant elements. For instance, N and S have been demonstrated to regulate the antioxidation potential of CNDs through several proposed mechanisms, such as hydrogen atom transfer or proton-coupled electron transfer, in various acidic media.^20,21^ Zhang et al. conducted a study of the radical scavenging potential of N, S codoped CNDs employing various techniques, including UV–vis-based and electrochemical assays. They concluded that a synergistic effect of both N and S as dopant elements could promote the radical scavenging abilities of these CNDs by providing greater electronegativity difference and defect production, respectively.^22^

Doping with P, given its bigger atomic size than that of C, could alter the electronic structure of CNDs.^23^ In a study by Li et al., an aggregation-induced red shift was observed in the fluorescence emission wavelength of P-doped CNDs.^24^ This phenomenon was explained by the fact that P atoms—as n-type donors—can enrich the electron density of the CNDs, and in combination with the size increment after aggregation, led to a π–π conjugation increment.^24^

The effect of B-doping, however, has been going through extensive controversies. Some studies have shown a reducing effect on the fluorescence intensity,^25^ while there are reports indicating the opposite.^26^ Some researchers believe that the difference between the size of B–C and C–C bonds can be considered a disorder that affects fluorescence.^27^ The charge transfer changes in the structure of N-doped CNDs upon doping them with P and B have been studied by attaching nickel(II) phthalocyanine to their surfaces.^28^ It was discovered that adding P to the CNDs accelerates the charge transfer process from carbon dots to phthalocyanine. B doping, on the other hand, has the opposite impact since the coupled hole transport is a slower process than electron transfer. Nonetheless, B-doping has been proven to promote charge transfer in other studies,^29^ driving more research in this area.

Although the influence of heteroatoms on the optical properties of carbon nanomaterials has been extensively studied, some key aspects of these effects on fluorescence emission and UV–vis absorption remain unclear. In the present work, we specifically investigate the effect of different levels of doped P and B on the optoelectronic and antioxidation properties of CNDs, which is known little and less studied previously. Finding a clear relationship among the electronic states, optical behavior, and radical scavenging capability of CNDs allows us to design superior CNDs, particularly for reactive oxygen species (ROS) scavenging applications.

## Materials and Methods

### Materials

Citric acid (CA), boric acid (BA), ethylenediamine (EDA), phosphoric acid (PA), quinine sulfate (QS), sulfuric acid (SA), 2,2-diphenyl-1-picrylhydrazyl (DPPH), and methanol were purchased from Sigma-Aldrich Chemie GmbH (Munich, Germany) and were used as received. All aqueous solutions were prepared using fresh deionized water. Dialysis bags were purchased from Sigma-Aldrich Chemie GmbH (Munich, Germany) and used for CND purification.

### Synthesis of CNDs

Based on the employed dopant element and the associated concentrations, five different CNDs were designed and synthesized, as summarized in Table 1. A one-step closed-vessel microwave method with similar conditions was used to synthesize all CNDs. The microwave method is a proven approach that prevents the formation of undesirable side products.^30^ Also, using EDA results in higher yields than other N-source precursors.^31^ Based on Table 1, the required quantities of each precursor were dissolved in deionized water and stirred until a clear solution was obtained. The solution was then transferred to a microwave synthesizer and heated up with proper setpoints (temperature = 180 °C, power = 300 Watts, and pressure = 100 Psi) for 30 min. After 30 min, the obtained brown solution was centrifuged at 4000 rpm for 20 min to precipitate large particles. The supernatant was further filtered using a 1 kDa dialysis membrane in deionized water for 48 h. Afterward, the product was freeze-dried, and the brown CND powder was collected, which was stored at room temperature for later use.

**Table 1:** Synthesis Design of Different CND Samples

sample name	CNDs type	P/B level	precursors			
			CA (g)	EDA (mL)	PA (mL) × 21.92 N^a^	BA (g)
E-CNDs	N, CNDs	0	1	1	0	0
low P%-CNDs	N, P CNDs	low P	1	1	0.5	0
high P%-CNDs	N, P CNDs	high P	1	1	2	0
low B%-CNDs	N, B CNDs	low B	1	1	0	0.23
high B%-CNDs	N, B CNDs	high B	1	1	0	0.95

a Normality.

### Characterization

The size measurement was conducted using atomic force microscopy (AFM, MFP-3D Origin, Asylum) by the height profiles.^32^ A 0.002 mg/mL solution of each CND sample was prepared in deionized water, sonicated for 20 min, drop-cast on a mica surface, and set aside to dry for AFM imaging. Fourier transform infrared spectroscopy (670 FTIR, Varian) was carried out to identify the formation of functional groups on the CNDs’ surface. Further elemental data analysis was carried out using X-ray photoelectron spectroscopy (ESCALAB 250Xi XPS, Thermo-Fisher). Solutions of 10 mg/mL of all CND samples were prepared, drop-cast, and dried out for XPS analysis. Dynamic light scattering (DLS, Malvern Instruments ZEN3600) was used to measure the zeta potential of CND samples with a concentration of 0.005 mg/mL. A spectrophotometer (XploRA ONE, Horiba) equipped with a coupled-charged device (CCD) camera was used to perform the Raman spectroscopy of CNDs, and a laser with 532 nm was used as the excitation light. The UV–vis absorption and the fluorescence spectrum of CND samples were measured using a UV–vis spectrophotometer (UV–Vis–NIR, Agilent) and a fluorometer (Fluoro-Max 4 fluorometer, Horiba), respectively.

### UV–vis Spectroscopy

All CND samples of 0.025 mg/mL solutions were prepared, and their UV–vis spectra were obtained and plotted to investigate the effect of added dopant heteroatoms on the absorbance. Also, to further evaluate the impact of doping on the band-gap energy, Tauc plots were studied using eq 1.^33^
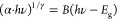
1where α, *h*, ν, *E*_g,_ and *B* are the absorption coefficient, Planck constant, photon frequency, band-gap energy, and the proportionality constant, respectively. In the calculation of the band-gap energy, we assumed that, due to the amorphous structure of CNDs, the direct electronic transition takes place upon the emission of UV light; therefore, the γ factor was considered 0.5. Once plotted, a tangent line was drawn on the curve in the area of 3–4 eV on the *X*-axis. The intersection point is considered as the apparent band-gap energy, which could be originating from the molecular-like HOMO and LUMO of the carbogenic core of CNDs.^33−35^

### Fluorescence and Quantum Yield (QY) Measurement

A 0.025 mg/mL solution of each CND sample was prepared, and the fluorescence emission spectrum was monitored in a wide range of excitation wavelengths. The effects of dopant heteroatoms on fluorescence emission intensity and the maximum wavelength shifts were investigated. Also, the relative QY was calculated with reference to QS as a standard at an excitation wavelength of 360 nm. A 0.1 mg/mL solution of QS in H_2_SO_4_ (0.1 M) was used as the reference. The integrated fluorescence of the CNDs at an excitation wavelength of 360 nm was calculated. The slope of fluorescence intensity over the maximum UV absorbance of each CND sample was calculated and compared to QS using eq 2.^36^
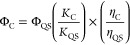
2where Φ is the QY, *K* is the slope determined from the curves, and η is the refractive index. The subscripts C and QS represent the CNDs and QS, respectively. It is noted that Φ_QS_ and  are considered to be 0.54 and 1, respectively.^36^

### Radical Scavenging Measurement of CNDs

As an established method of evaluating the antioxidation capability of CNDs, DPPH radical scavenging assay was used in which the UV–vis absorbance change of DPPH radical solution was monitored at 517 nm upon the addition of CNDs.^37,38^ Different CND concentrations were prepared in deionized water, incubated with a solution of DPPH (0.02 mg/mL in methanol), and mixed simultaneously under dark conditions for 2 h. Finally, the UV–vis absorbance of each solution was measured at 517 nm. The antioxidation activity% of CND samples was calculated using eq 3.^20,21^
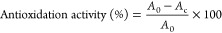
3where *A*_0_ and *A*_c_ are the absorbances of the DPPH radical at 517 nm in the absence and presence of CNDs, respectively.

## Results and Discussion

### Synthesis and Characterization of CNDs

The closed-vessel microwave approach was chosen for the synthesis to obtain a satisfactory B-doped product. Once the synthesis process for producing B-doped CNDs was optimized, the synthesis procedures of other CNDs were adjusted accordingly. The yield of synthesis reactions of E-CNDs, Low P%-CNDs, High P%-CNDs, Low B%-CNDs, and High B%-CNDs was 2.5, 4.42, 1.73, 5.38, and 3.38%, respectively.

AFM results showed that all CNDs were spherical with an average size of 2.31 ± 0.28 nm for E-CNDs, 0.43 ± 0.04 nm for Low P%-CNDs, 0.75 ± 0.07 nm for High P%-CNDs, 0.2 ± 0.01 nm for Low B%-CNDs, and 0.81 ± 0.11 nm for High B%-CNDs (Figure S1). An ordinary one-way analysis of variance (with a *P* value of 0.05) shows that only the size of E-CNDs is significantly larger and different from the heteroatom P or B-doped CNDs; the changes in the particle size between low and high P or B dopant CNDs are not significant.

**Figure 1:**
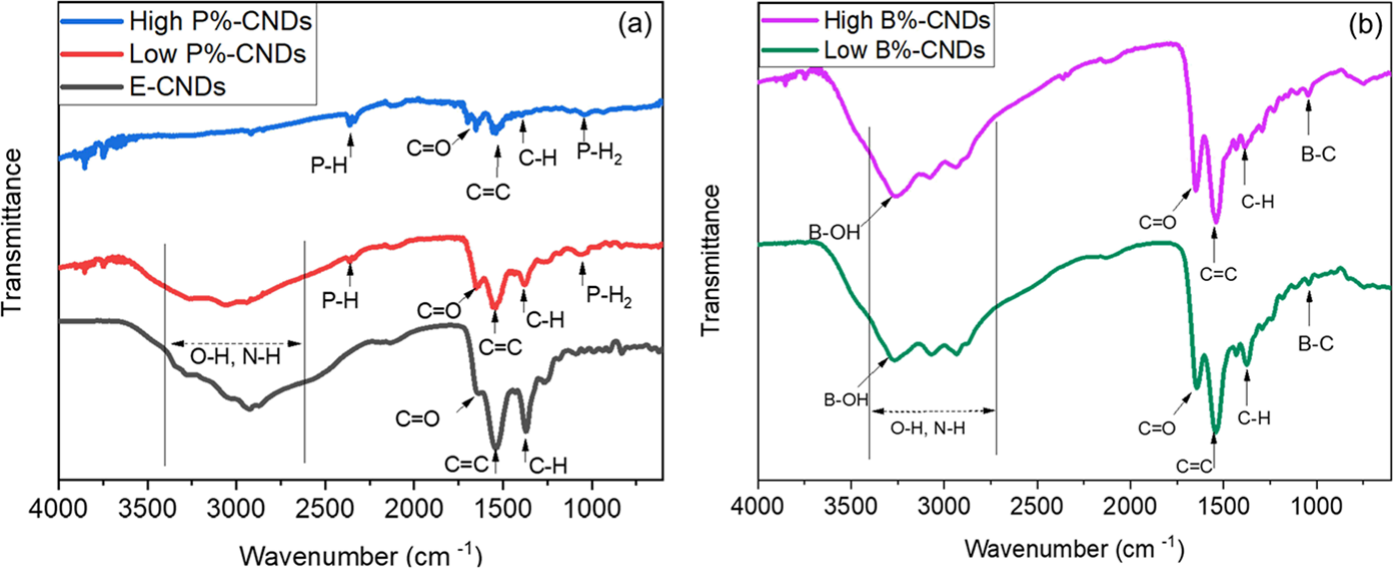
(a) FTIR results of E-CNDs, Low P%-CNDs, and High P%-CNDs, and (b) Low B%-CNDs, and High B%-CNDs.

Figure 1 shows the FTIR spectra of the synthesized CNDs. The C=O stretch at 1650 cm^–1^ and the O–H stretch at 3100 cm^–1^ in the FTIR spectra confirm the presence of the COOH group in all CNDs except the High P%-CNDs.^39^ The observed peak around 1550 cm^–1^ can be attributed to C=C of the graphitic structure of the CND core.^40^ In all CNDs, the N–H bonds appeared around 3300–3350 cm^–1^ (amine N–H). In both P-doped CNDs, the peaks that appear at 1040 and 2330 cm^–1^ are assigned to P–H_2_ and P–H bonds, respectively.^19^ In the case of B-doped CNDs, the band that appears at 1000 cm^–1^ is assigned to B–C chemical bonds, and the band that appears at 3268 cm^–1^ is assigned to B–OH bonds.^41^ However, the broad peak at 2600–3400 cm^–1^ corresponding to O–H and N–H bonds, observed in E-CNDs, Low P%-CNDs, Low B%-CNDs, and High B%-CNDs, was not shown in the High P%-CNDs’ FTIR spectrum. This can be explained by the dehydration effect of the high concentration of phosphoric acid used in the synthesis of High P%-CNDs.

**Figure 2:**
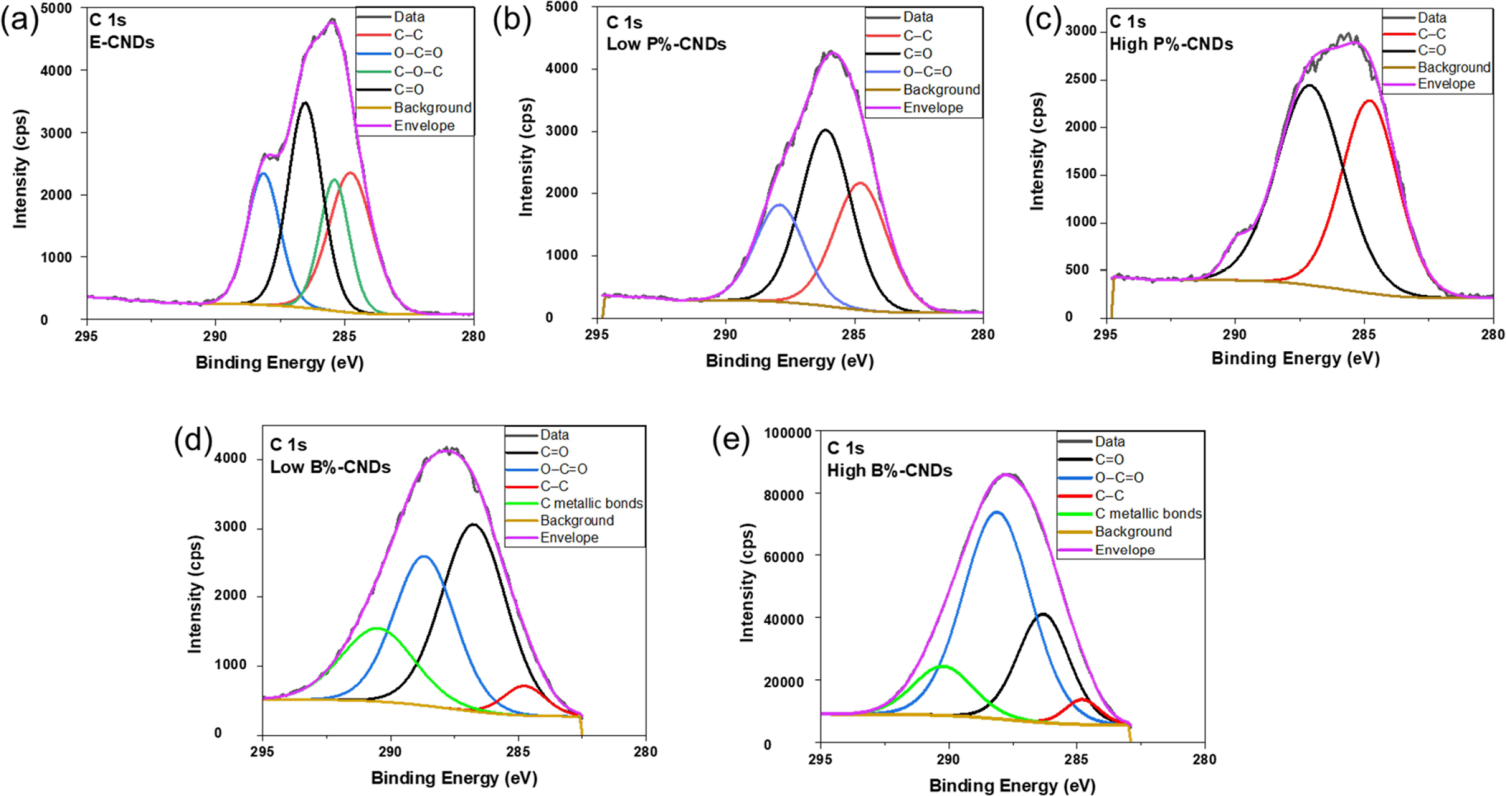
XPS analysis of C 1s scan of (a) E-CNDs, (b) Low P%-CNDs, (c) High P%-CNDs, (d) Low B%-CNDs, and (e) High B%-CNDs.

The full XPS survey of all CND samples is presented in Figure S2; Figure 2 shows the XPS spectra for C 1s; Figure S3a–g for P 2p, B 1s, and N 1s. Table S1 summarizes the XPS data analysis for the breakdown atomic percentage of P and B for each CND sample. In the analysis of E-CNDs, the P and B quantities were detected as trace levels of 0.04% and zero, respectively. The Low and High P%-CNDs contain 0.38 and 4.47% phosphorus, while the Low and High B%-CNDs have 0.5 and 1.95% boron, respectively. The analysis of the C 1s scan of CND types (see Figure 2 and Table 2) shows that C–C and C=O chemical bonds have been detected in all CND types, with the highest percentage belonging to High-P% CNDs for both bonds. On the other hand, the O–C=O chemical bond has not been detected in High-P% CNDs,^42^ while detected in B-CNDs.^29^ The C–O–C chemical bond has only been detected in the structure of E-CND. Only in the case of High-B% CNDs, peaks assigned to the metallic bonds of C have been observed.

**Table 2:** Percent of Detected Carbonic Chemical Bonds by XPS

assigned chemical bond (%)	E-CNDs	low P%-CNDs	high P%-CNDs	low B%-CNDs	high B%-CNDs
C–C	28.61	32.93	43.99	4.43	3.66
O–C=O	20.31	23.35		32.91	59.87
C–O–C	18.09				
C=O	33	43.72	53.82	43.44	23.92
metallic bonds with carbon				19.22	12.56

From Figure S3a,b and Table S2, one can find that in Low P%-CNDs, all P atoms are present in the form of P–C bonds, while the P–O and P–N bonds in addition to P–C bonds begin to form in High P%-CNDs.^28,43,44^ From Figure S3c,d and Table S3, it can be seen that in Low B%-CNDs, the primary presence of B is mainly binding with the functional groups of the C, i.e., −COO. In High B%-CNDs, B is more likely to form chemical bonds with CND’s surface carbon atoms as well,^26,28,29,41,45^ suggesting that B atoms might have less penetration capability to the carbogenic core of CNDs. In the B doping C 1s scans, two additional peaks also appeared at around 290 eV which can be assigned to metal carbonates.^46^

DLS was conducted to further investigate the effect of doping on the surface zeta potential (ζ) of CNDs, and the results are shown in Figures S4a–e and S5a. The carboxylic acid group is known to be the dominant source of negative charge on the surface of CNDs.^47^ In P dopant CNDs, a positive surface charge shift was found where the High P%-CNDs (ζ = −9.8 mV) and Low-P%-CNDs (ζ = −21.1 mV) present less negative charges compared to E-CNDs (ζ = −27.6 mV). In contrast, more negative surface charge of the B dopant CNDs was found as ζ = −34.1 mV for High B%-CNDs and ζ = −28.1 mV for Low B%-CNDs which can be attributed to the high percentage of O–C=O chemical bonds (59.87%). With the decrease of the O–C=O chemical bond portion, the surface charge of Low B%-CNDs and High P%-CNDs tends to have less accumulated negative charge. The results are corroborated by the percentage of O–C=O bonds found in different dopant CNDs in Table 2.

To further evaluate the potential effect of doping on the internal graphitic structure of CNDs, Raman spectroscopy was carried out. The G-band represents the sp^2^ hybridization of C atoms in their planer geometry, while the D-band represents the presence of dangling C atoms with the sp^3^ hybridization.^48^ After baseline subtraction, the Raman spectra were plotted for E-CNDs, High P%-CNDs, and High B%-CNDs (Figure S6). As expected, the D and G bands were observed at 1332 and 1585 cm^–1^, respectively. The degree of disorder—induced by P and B doping—was calculated using the *I*_D_/*I*_G_ ratio and was found to be 0.91, 0.73, and 0.69 for E-CNDs, High P%-CNDs, and High B%-CNDs, respectively (Figure S5b). It was observed that E-CNDs possess the highest *I*_D_/*I*_G_ ratio among all samples, which is due to the presence of defective moieties in their structure.^49^ The High P%-CNDs and High B%-CNDs, on the other hand, have lower *I*_D_/*I*_G_ ratios. The observed difference in this parameter can be attributed to the interference of N, P, and B in the carbogenic structure of CNDs during synthesis. As summarized in Table S4, the bond length of C–N is much different (17.46%) than C–C while the lengths of C–P (9.85%) and C–B (4.92%) are less different. Therefore, the effect of C–B defects on the carbonic core of CNDs is less than those from C–P and C–N while the C–N has the largest effect on forming defective structures.^50^

### Optical Property Studies

**Figure 3:**
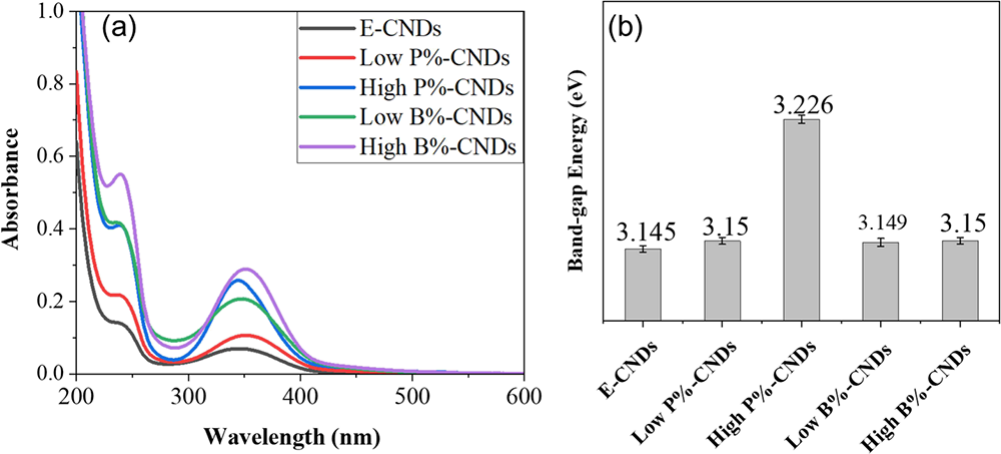
(a) UV–vis spectra and (b) calculated band-gap energies of CND samples.

CNDs usually exhibit two significant UV–vis absorbance peaks.^13^ The first is a shoulder peak near 240 nm related to π–π* transitions of C=C bonds originating from the carbogenic cores. The second one is a strong, broad peak near 345 nm, attributed to n−π* transitions of nonbinding orbitals mostly belonging to C=O bonds on the surface of CNDs. Hence, the latter is more affected by the functional groups and dopant heteroatoms.^19^Figure 3a exhibits the UV–vis spectrum of all synthesized CNDs at 0.025 mg/mL. Both B and P dopants and the doping level increase UV–vis absorbance. This can be explained by the formation of abundant functional groups on the surface of CNDs, i.e., P–O and P–H of P-CNDs, and B–N and B–OH of B-CNDs (see Figure 1). Since N has larger electronegativity (3.04) than P (2.19) and B (2.04), electrons of functional groups involving N are less likely to get excited compared to the electrons of functional groups involving P or B. Based on this, one could conclude that the availability and feasibility of n−π* transitions would be in the order of B-CNDs > P-CNDs > E-CNDs.

From the UV–vis absorbance spectra, Tauc plots were obtained to estimate the energy band gap (Figure S7). It was found that only the High P%-CNDs has a significant increase of band gap to 3.23 eV from around 3.15 eV of all other types of CNDs. It is expected to observe a lower energy band gap in High P%-CNDs, while surprisingly, it increased significantly. A similar observation was reported by Sekar et al.^51^ This can be attributed to the presence of more C=O chemical bonds (Table 2) which play a key role in strengthening the n−π* transition. Higher electronegativity of P compared to B causes an increase in the n−π* transition energy gap, which is also apparent as a shift in the XPS peak, i.e., 287.1 vs 286 eV for C=O in High P%-CNDs and High B%-CNDs, respectively.

In the case of the B dopant, the energy gap change is insignificant, a good agreement with the fact that the effect of B doping on the functional groups is more dominant compared to that on penetration discussed earlier. It is consistent with the effect of functional groups on decreasing the HOMO and LUMO.^52^ As a result, the maximum absorption of High P%-CNDs has blue-shifted. This blue shift is due to the higher energy required for the electron transition between the HOMO and LUMO.^33^

**Figure 4:**
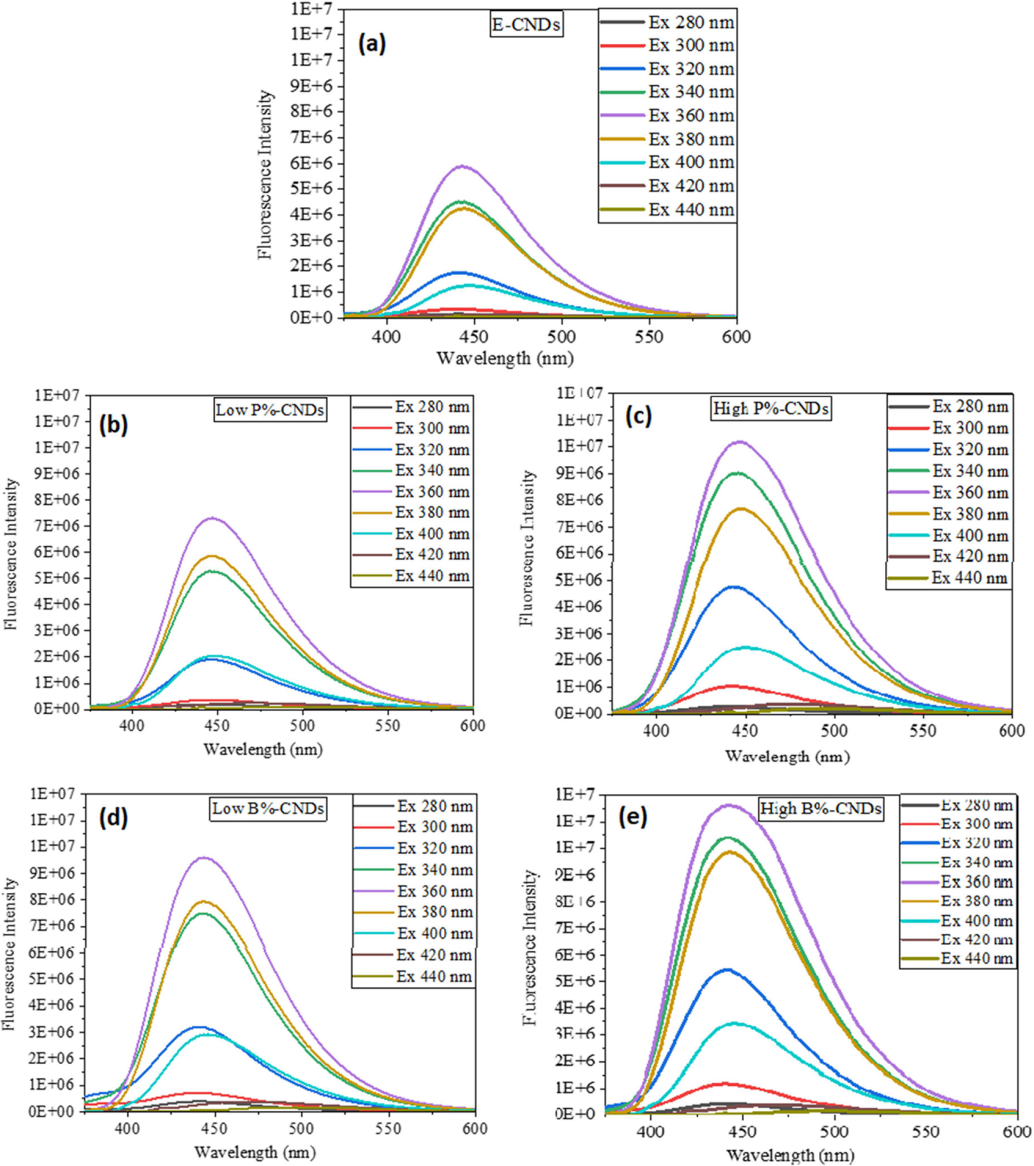
Fluorescence spectra of (a) E-CNDs, (b) Low P%-CNDs, (c) High P%-CNDs, (d) Low B%-CNDs, and (e) High B%-CNDs.

The fluorescence spectra of all CND samples were examined at different excitation wavelengths (Figure 4). The most significant fluorescence emission was observed at a wavelength of 360 nm in all samples. Unlike the emission intensity—which varied considerably across all samples—the maximum emission wavelength variation was insignificant (443 nm for E-CNDs and B-doped CNDs, 447 nm for P-doped CNDs). Also, in all cases, excitation-independent fluorescence emission was observed. The addition of P boosted the fluorescence intensity by providing additional electrons as an n-type donor, as seen in Figure S8. However, compared to P, the addition of B dopant led to a more significant enhancement in the fluorescence intensity. It is believed that higher fluorescence usually originates from less defects, while low/medium fluorescence originates from having more defects in CNDs.^53^ Since the B dopant has shown less penetration to the core, B-doped CNDs are assumed to have more ordered core than P dopant CNDs, thus higher fluorescence yield. This analysis is in a good agreement with the measured fluorescence and Raman results, i.e., the higher fluorescence intensity of B-doped CNDs over others.

The QYs of all CNDs were calculated using QS as a reference (Figure S9a). Figure S9b shows the QYs of all synthesized CNDs. Although codoping is expected to enhance the QY of CNDs due to the synergistic impact of trapped electron–hole pairs of dopants,^14^ such a trend was not observed in P and B dopants. Surprisingly, the calculated QY trend of CNDs did not match their fluorescence intensity order. With the addition of P to CNDs, the QY was observed to decline, with similar observation in the case of B doping. This can be explained by the fact that QY is calculated using both the light absorbances and emission intensities of each CND. Because doping affects the UV–vis absorption properties of CNDs differently than fluorescence, it is not surprising that the QY fluctuated. Another plausible explanation for the QY results is that the attained QY is dependent on the balance of the surface −COOH and −OH groups. This balance may have been disrupted in various ways during doping CNDs with P and B due to the formation of additional functional groups on the surface.^53^

**Figure 5:**
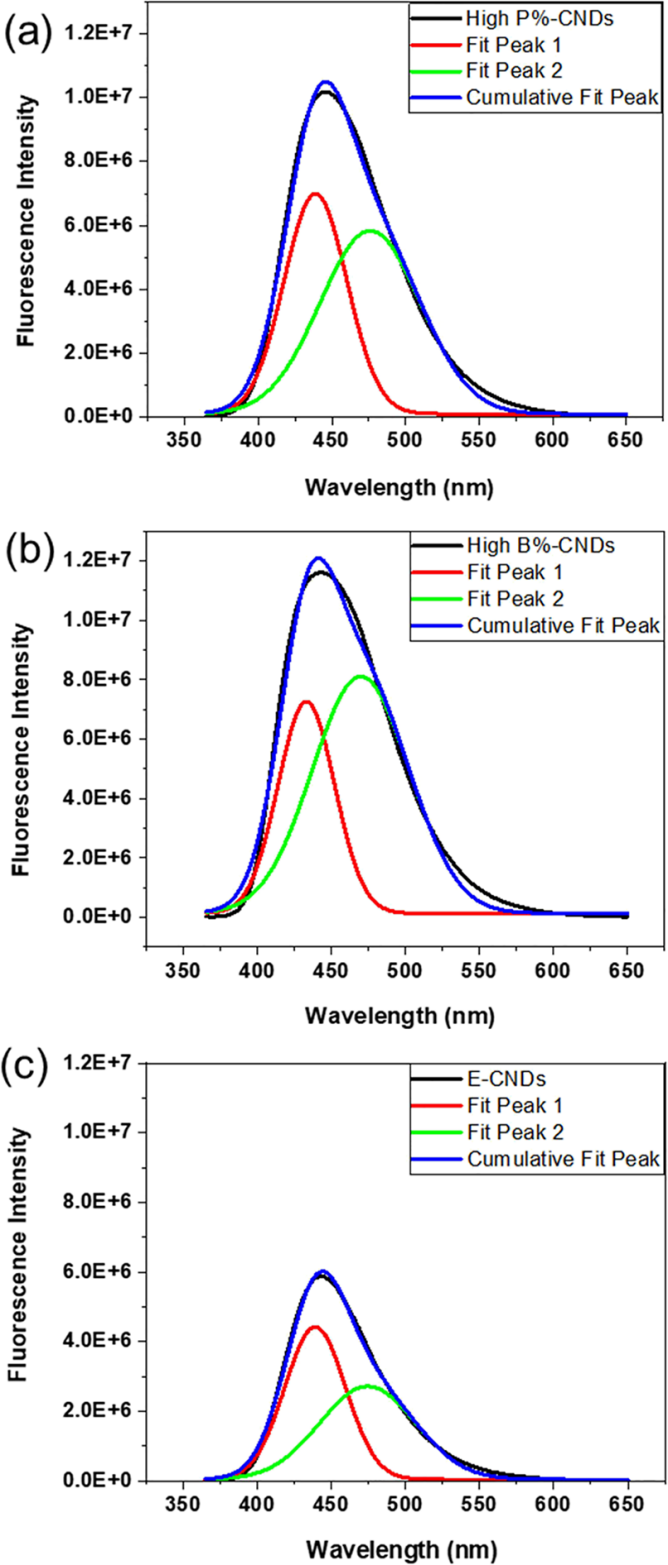
Fluorescence deconvolution graphs for (a) High P%-CND, (b) High B%-CND, and (c) E-CNDs samples.

Deconvoluting the fluorescence peaks of CNDs is a technique that aids in determining the origin of the fluorescence. The emission peak of CNDs is deconvoluted into two Gaussian sub-peaks, namely, fit peak 1 and fit peak 2, that reflect the fluorescence caused by sp^2^ defects and the surrounding functional groups, respectively.^54,55^Figure 5a–c and Table S5 demonstrate the relative dominance of the sub-peaks as well as their full width–half maximum (FWHM) ratios. Figure 5a shows that in the High P%-CNDs, fit peak 1 is dominant, indicating that sp^2^ defects have more impact on the fluorescence than the functional groups. The reverse effect is seen in High B%-CNDs, where fit peak 2 has a more considerable dominance, corresponding to the stronger effects of functional groups on fluorescence. In addition, the FWHM ratio (Table S5) of fit peak 1 to fit peak 2 is calculated as 0.62 and 0.58 for High P%-CNDs and High B%-CNDs, corresponding to the dominance of sp^2^ defects or surface functional groups, respectively. In E-CNDs with N as the dopant heteroatom, the fit peak 1 dominates the fit peak 2, and the ratio of FWHM of the fit peak 1 to fit peak 2 is larger than the high P% and high B% CNDs. This outcome agrees with the Raman data which indicates that N plays a significant role causing defects in the carbonic core due to the length of the N–C bond.

On the other hand, the different effects of P and B dopants on the surface functional groups to some extent account for the fluorescence intensity. Since the p*K*_a_ of phosphoric acid^56^ is lower than that of boric acid,^57^ it is more likely that the OH groups of phosphoric acid undergo deprotonation than the OH groups of boric acid. Hence, there could be more OH groups available on the surface of B-doped CNDs during the synthesis. As electron donors, the OH groups can suppress nonradiative processes and improve the integrity of the π conjugated system.^58,59^ This can contribute to the increased fluorescence of B-doped CNDs. Furthermore, as discussed above, the surface states are primarily responsible for the fluorescence of B-doped CNDs, and their fluorescence was observed to be higher than that of P-doped CNDs, one can conclude that the surface chemistry of CNDs plays a more significant role in the origin of fluorescence than the defects induced in their graphitic core to the P and B doped CNDs. It is worth noting that the above discussed findings provide a guidance to potentially tune the overall fluorescence wavelength to red or near-infrared regions if introducing more surface functional groups by higher B% doping and more π conjugated structures such as C=C, C=N/C=O, a good agreement with previous studies.^60^

Further analysis of XPS data corroborates the observation of high fluorescence in high B% CNDs. In the N 1s XPS scan of E-CNDs and High P%-CNDs (Figure S3e,f), only one peak was observed at ∼400.2 eV which is attributed to −NH_2_ bonding. In the N 1s XPS scan of High B%-CNDs (Figure S3g), another peak appeared at ∼403 eV, which can be assigned to NO_3_^–^ bonding. It has been shown that CNDs with fewer amino groups on the surface have higher fluorescence than amino-rich CNDs because of the surface passivation with the presence of NH_2_ groups.^23,61^ On the other hand, the NO_3_^–^ functional group is capable of strengthening the fluorescence by providing π–π conjugations through the electron resonance among N–O bonds in its structure.^62^

### Radical Scavenging Studies

**Figure 6:**
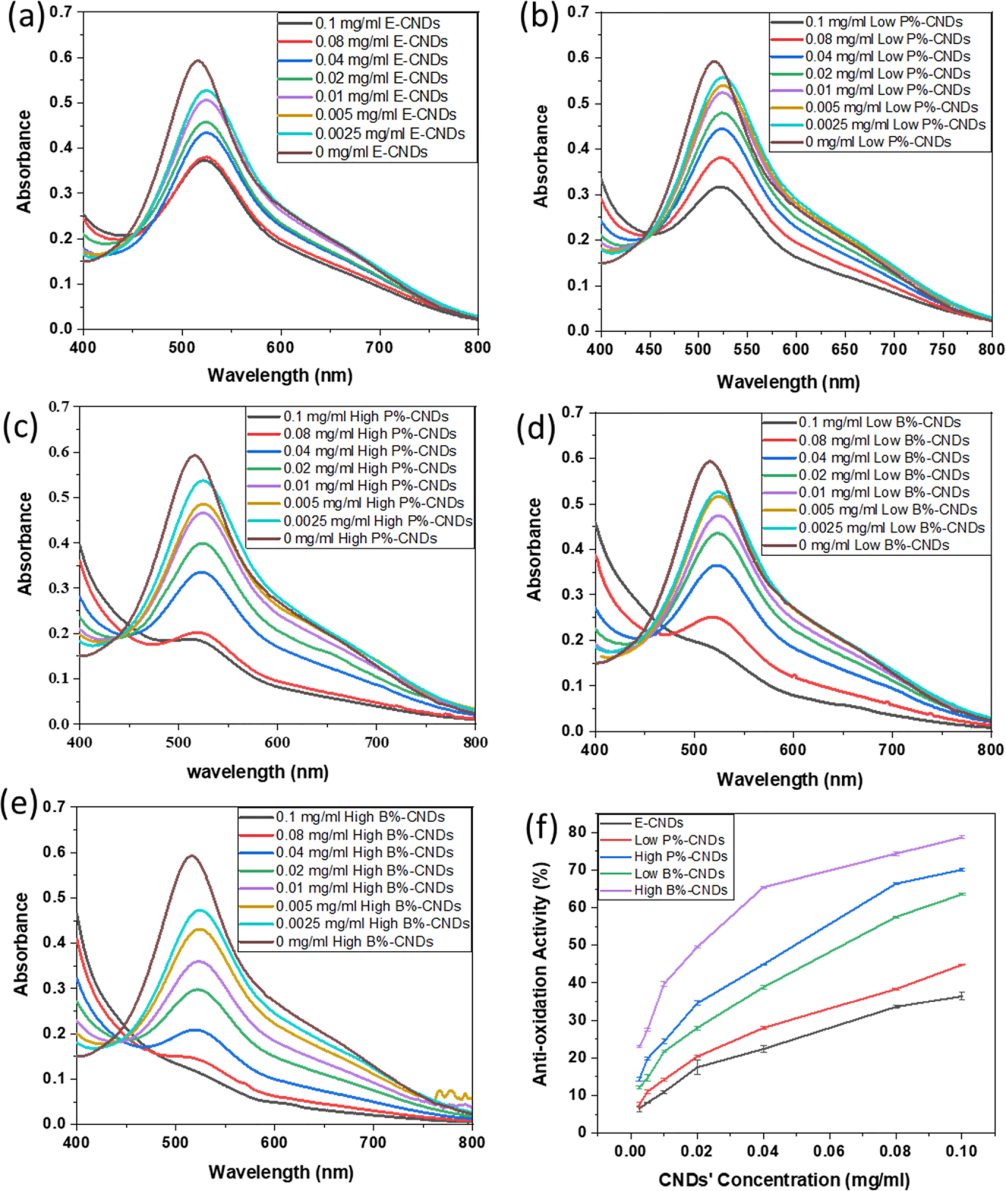
UV–vis absorbance spectra of DPPH radicals (0.02 mg/mL) at different concentrations of CNDs for (a) E-CNDs, (b) Low P%, (c) High P%, (d) Low B%, (e) High P%, and (f) percentage of antioxidation activities of CND samples.

CNDs showed as promising candidates for ROS scavenging in cellular studies.^63^ The UV–vis absorbance spectra of DPPH radicals (0.02 mg/mL) at different concentrations of CNDs were obtained and are shown in Figure 6a–e. As the concentration of CNDs increases, the absorbance intensity falls, showing their radical scavenging capabilities. Figure 6f illustrates the antioxidation activity (%) of different CNDs vs their concentration calculated by eq 3. Compared to E-CNDs, the P or B-doped CNDs presented higher levels of anti-oxidation activities. Specifically, the antioxidation activity (%) increased with the increase of the P or B where the High B%-CNDs exhibited 78.68% antioxidation activity, being superior to the other CNDs. The order of the antioxidation capability was found to be High B% > High P% > Low B% > Low P% > E-CNDs.

**Scheme 1:**
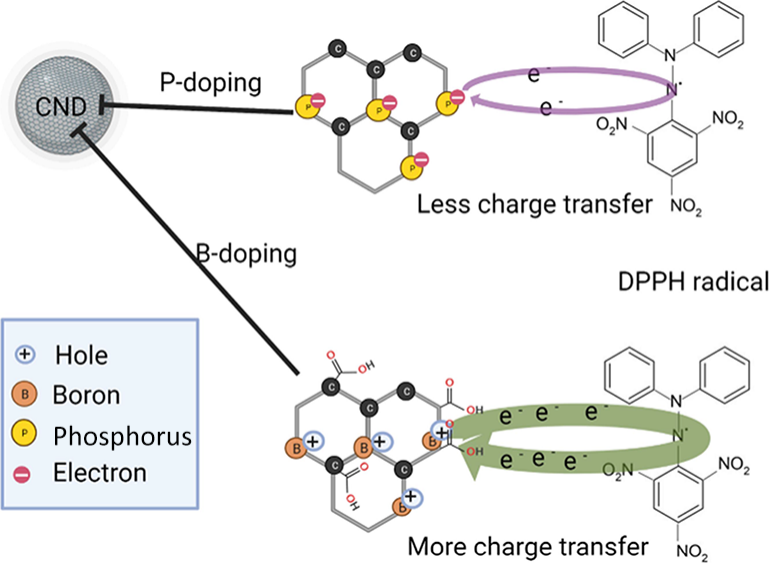
Proposed DPPH Radical Scavenging Mechanism for the P- and B-Doped CNDs, and the Enhanced Scavenging Capability of High B%-CNDs

This performance can be attributed to the transfer of both electrons (induced by n-type N doping) and holes (related to boron p-type doping) during the interactions between DPPH and CNDs. According to XPS analysis, High B%-CNDs have the highest percentage of O–C=O in their structure, which can act as a hydrogen donor, donating a hydrogen ion to a free radical species and neutralizing it.^64^ Additionally, compared to P-doped CNDs, XPS data confirmed that by adding more B to the CNDs’ structure, the percentage of C–C (sp^3^ hybridization) decreased significantly. As a result, it is expected that there is a higher probability of carbon sp^2^ hybridization and thus high possibility of electron transfer between the High B%-CNDs and DPPH. As a summary, Scheme 1 depicts the discussed radical scavenging mechanisms of the P and B-doped CNDs and the enhanced scavenging capacity of High B%-CNDs.

## Conclusions

This research focuses on the changes in optical characteristics of different CNDs with varied levels of P and B as dopant heteroatoms. Analysis of DLS, Raman, and XPS data combining with UV–vis absorbance and fluorescence of the CNDs demonstrate that both P and B affected the optoelectronic properties of CNDs, albeit in different respects. XPS results showed that P, although in trace quantities, showed a higher penetration ability into the carbonic core of CNDs. In contrast, B predominantly formed chemical bonds at the surfaces of CNDs, and it was only after raising the B level that additional chemical bonds involving the carbonic core were likely developed. The findings were further confirmed by Raman data analysis, which showed that the *I*_D_/*I*_G_ for High P%-CNDs was greater than that of High B%-CNDs. In terms of the band-gap analysis, however, High P%-CNDs turned out unexpectedly to be larger than the other CNDs. This result was attributed to the presence of C=O chemical bonds, which may intensify n−π* transitions and thus increase the band-gap energy. Further deconvolution of the fluorescence spectrum indicated that the dominant emission originating from the carbogenic core of High P%-CNDs was more significant than that in High B%-CNDs. As a conclusion, the P dopant as an n-type donor can boost the fluorescence of CNDs by influencing the carbonic core and electron–hole radiative recombination, while the B dopant as a p-type donor can improve the fluorescence by more significant interference to the surface functional groups of more emissive trap states. The DPPH radical scavenging study suggests that the B dopant had a greater influence on the scavenging capability of CNDs than the P dopant, most likely due to more O–C=O bonds and fewer C–C bonds in High B% CNDs, which promotes favorable charge transfer between the CNDs and DPPH. This research may offer a guidance in designing the structures of CNDs in the core and surface functional groups as an effective method for tuning their optoelectronic properties for photoluminescence and antioxidation applications.

## Supporting Information Available

AFM images of CNDs, XPS data, zeta potential and analysis, Raman spectra of CNDs and analysis, UV–vis absorbance spectra and energy gap, fluorescence analysis, and quantum yield (PDF)
