# Supplementary material for: Effect of Doping Heteroatoms on the Optical Behaviors and Radical Scavenging Properties of Carbon Nanodots
Source: J Phys Chem C Nanomater Interfaces. 2023 Apr 5;127(15):7360–70. doi: 10.1021/acs.jpcc.3c00953 (PMC10123816; doi:10.1021/acs.jpcc.3c00953)
Supplement: Supplementary file 1 — jp3c00953_si_001.pdf [file jp3c00953_si_001.pdf]

Supporting Information

Effect of Doping Heteroatoms on The Optical Behaviors and Radical Scavenging Properties of Carbon Nanodots

Mahsa Azami,[†] *Jianjun Wei*[*,†] *Mehrab Valizadehderakhshan,*[‡] *Anitha Jayapalan,*[†] *Olubunmi O Ayodele,*[†] *Kyle Nowlin*[†]

[†]*Department of Nanoscience, Joint School of Nanoscience and Nanoengineering (JSNN), University of North Carolina at Greensboro, Greensboro, NC 27401, USA.*

[‡] *Joint School of Nanoscience and Nanoengineering (JSNN), North Carolina Agricultural and Technical State University, Greensboro, NC 27401, USA.*

[*]Corresponding Author E-mail: j_wei@uncg.edu

Table of Content

Figure S1. AFM results of E-CNDs (a), Low P%-CNDs (b), High P%-CNDs (c), Low B%-CNDs (d), High B%-CNDs (e). and ANOVA analysis of size difference (f). Pg S3-S4

Figure S2. XPS survey analysis of E-CNDs (a), Low P%-CNDs (b), High P%-CNDs (c), Low B%-CNDs (d), and High B%-CNDs (e). Pg S5

Figure S3. XPS scan of P 2p in Low P% CNDs (a), P 2p in High P% CNDs (b), B 1s in Low B% CNDs (c), B 1s in High B% CNDs (d), N 1s in E-CNDs (e) N 1s in High P%-CNDs (f), and N 1s in High B%-CNDs (g). Pg S6-S7

Figure S4. DLS graph of E-CNDs (a), Low P%-CNDs (b), High P%-CNDs (c), Low B%-CNDs(d), and High B%-CNDs (e). Pg S8-S9

Figure S5. Zeta potential values of different CNDs samples (a), and ID/IG ratios of E-CNDs, High P%-CNDs, and High B%-CNDs (b). Pg S10

Figure S6. Raman spectra of E-CNDs, High P%-CNDs, and High B%-CNDs Pg S10

Figure S7. Tauc plots representing the band-gap energy of CND samples. Pg S11

Figure S8. Fluorescence intensity of CND samples. Pg S12

Figure S9. QY of CND samples compared to that of QS (a) and calculated QY of CND samples (b). Pg S12

Table S1. Percentage of dopant elements across different CND samples extracted from XPS results. Pg S13

Table S2. Atomic percentage of detected phosphorous chemical bonds by XPS. Pg S13

Table S3. Atomic percentage of detected boron chemical bonds by XPS. Pg S14

Table S4. I_D/I_G ratios and bond lengths in different CND samples. The bond length data were obtained from National Institute of Standards and Technology data base. Pg S14

Table S5. Dominance and ratios of fit peaks' FWHM for E-CNDs, High P%-CNDs, and High B%-CNDs. Pg S15

Reference. Pg 15

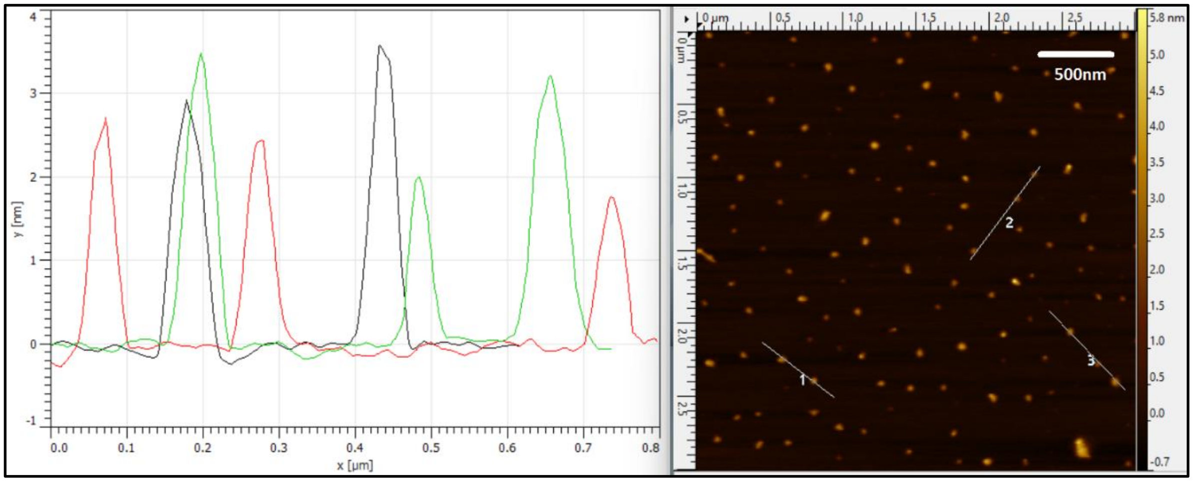
y [nm]
x [µm]
500nm
5.8 nm
1
2
3
(a)

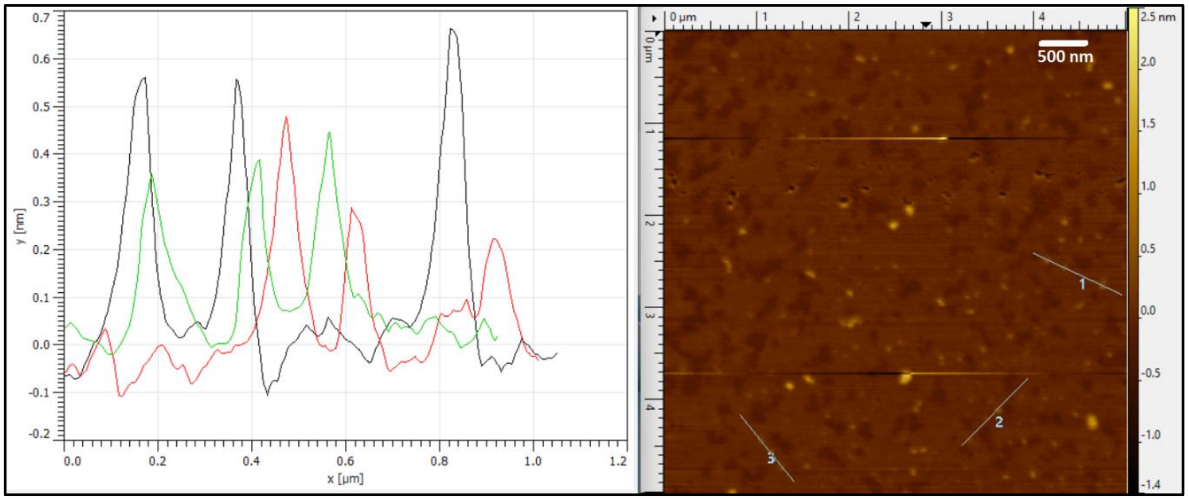
y [nm]
x [µm]
500 nm
2.5 nm
1
2
3
(b)

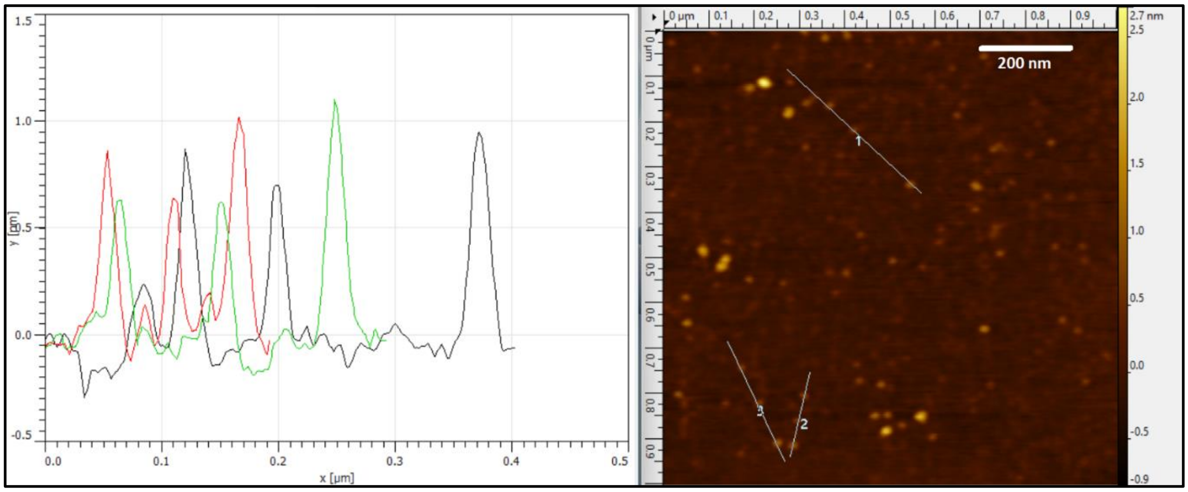
y [nm]
x [µm]
200 nm
2.7 nm
1
2
3
(c)

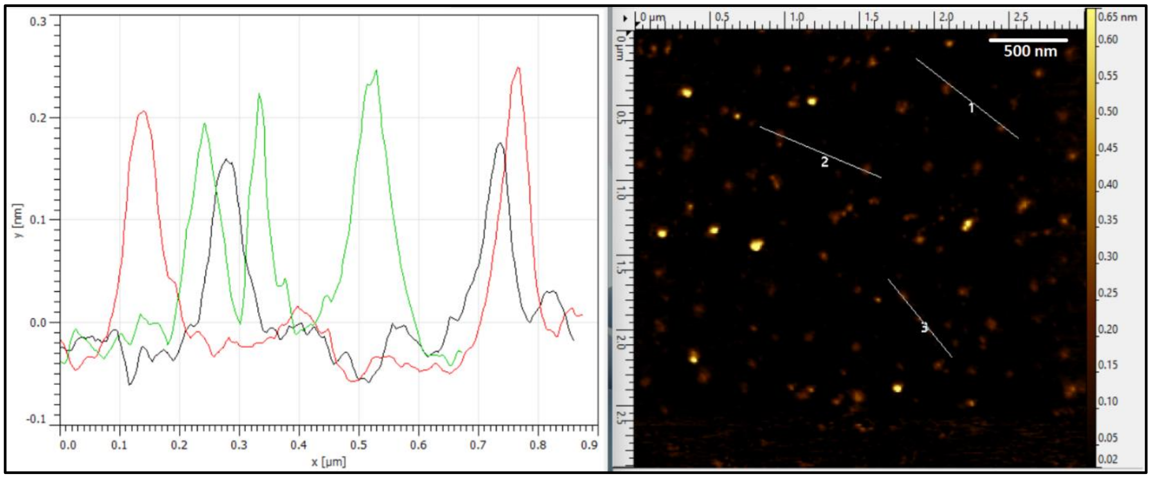

(d)

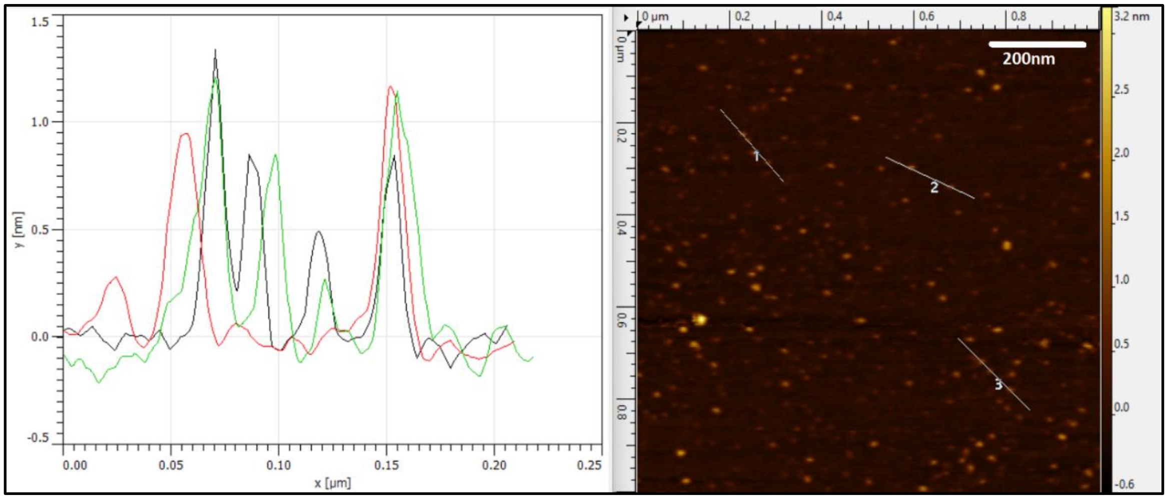

(e)

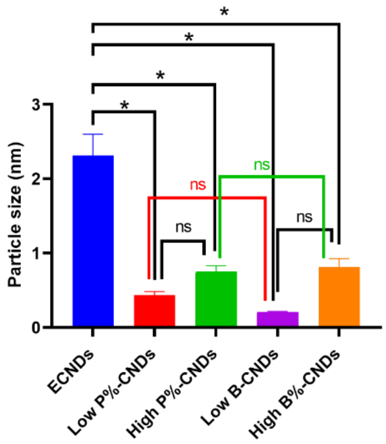

(f)

Figure S1. AFM results of E-CNDs (a), Low P%-CNDs (b), High P%-CNDs (c), Low B%-CNDs (d), High B%-CNDs (e), and ANOVA analysis of size difference (f).

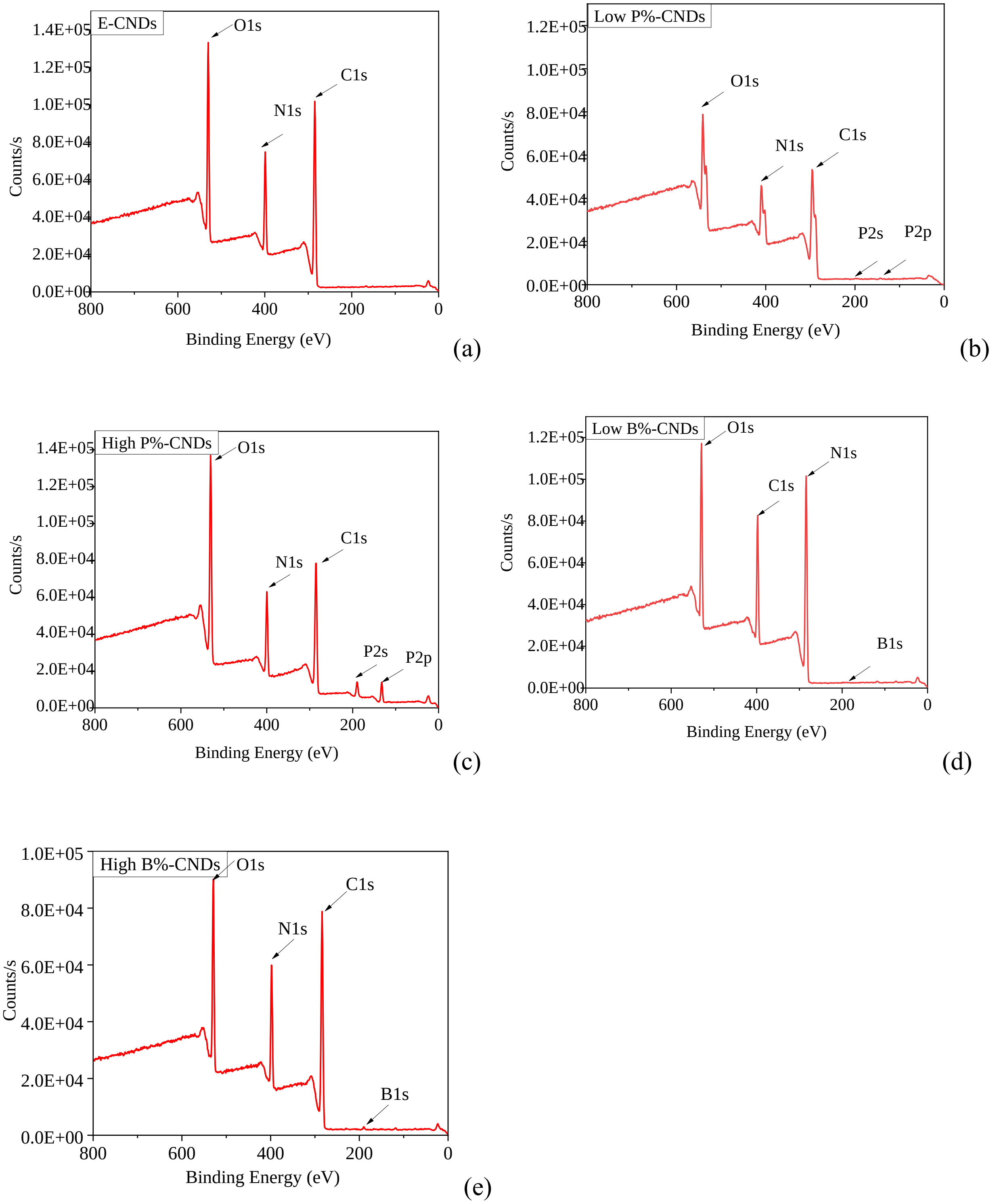

Figure S2. XPS survey analysis of E-CNDs (a), Low P%-CNDs (b), High P%-CNDs (c), Low B%-CNDs (d), and High B%-CNDs (e).

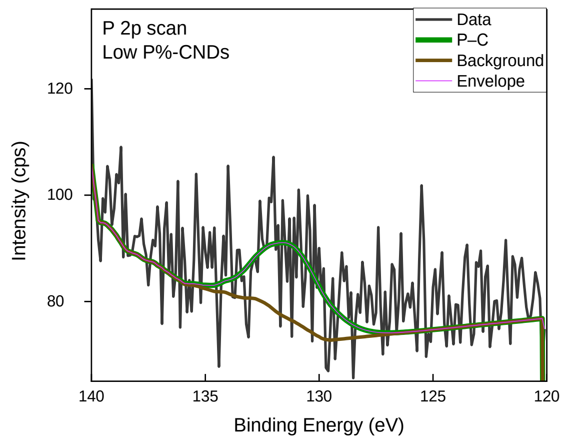
P 2p scan
Low P%-CNDs
Data
P–C
Background
Envelope
Intensity (cps)
Binding Energy (eV)
120
100
80
140
135
130
125
120

(a)

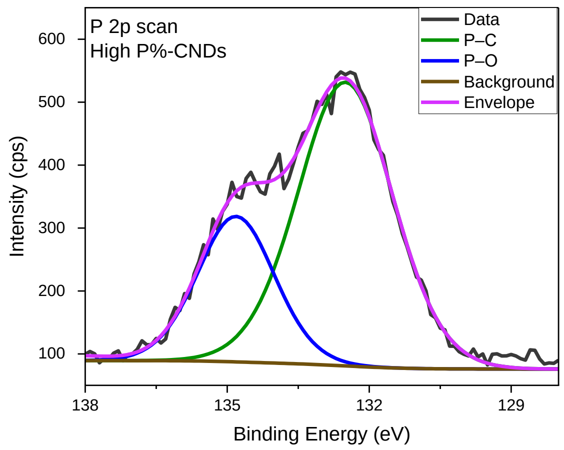
P 2p scan
High P%-CNDs
Data
P–C
P–O
Background
Envelope
Intensity (cps)
Binding Energy (eV)
600
500
400
300
200
100
138
135
132
129

(b)

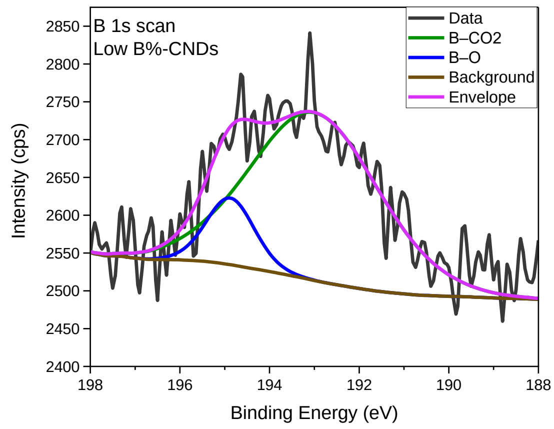
B 1s scan
Low B%-CNDs
Data
B–CO2
B–O
Background
Envelope
Intensity (cps)
Binding Energy (eV)
2850
2800
2750
2700
2650
2600
2550
2500
2450
2400
198
196
194
192
190
188

(c)

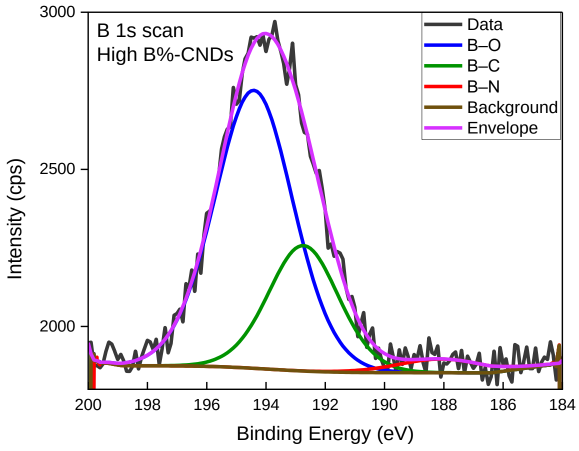
B 1s scan
High B%-CNDs
Data
B–O
B–C
B–N
Background
Envelope
Intensity (cps)
Binding Energy (eV)
3000
2500
2000
200
198
196
194
192
190
188
186
184

(d)

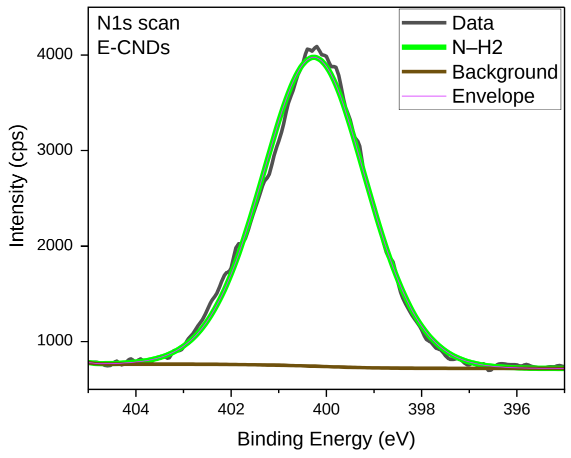
N1s scan
E-CNDs
Data
N–H2
Background
Envelope
Intensity (cps)
Binding Energy (eV)
4000
3000
2000
1000
404
402
400
398
396

(e)

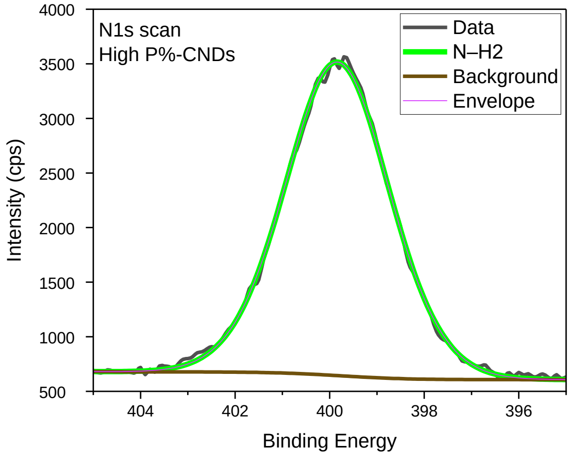
N1s scan
High P%-CNDs
Data
N–H2
Background
Envelope
Intensity (cps)
Binding Energy
4000
3500
3000
2500
2000
1500
1000
500
404
402
400
398
396

(f)

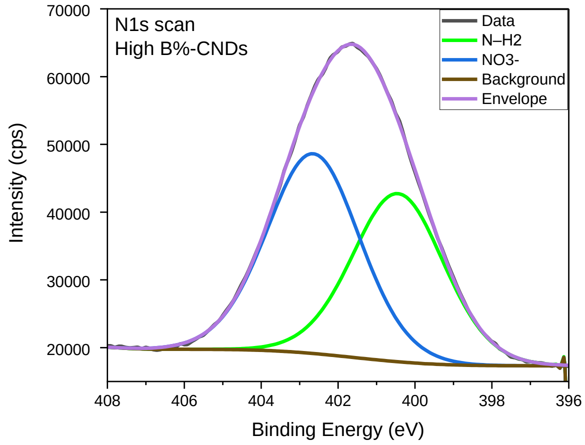

(g)

Figure S3. XPS scan of P 2p in Low P% CNDs (a), P 2p in High P% CNDs (b), B 1s in Low B% CNDs (c), B 1s in High B% CNDs (d), N 1s in E-CNDs (e) N 1s in High P%-CNDs (f), and N 1s in High B%-CNDs (g).

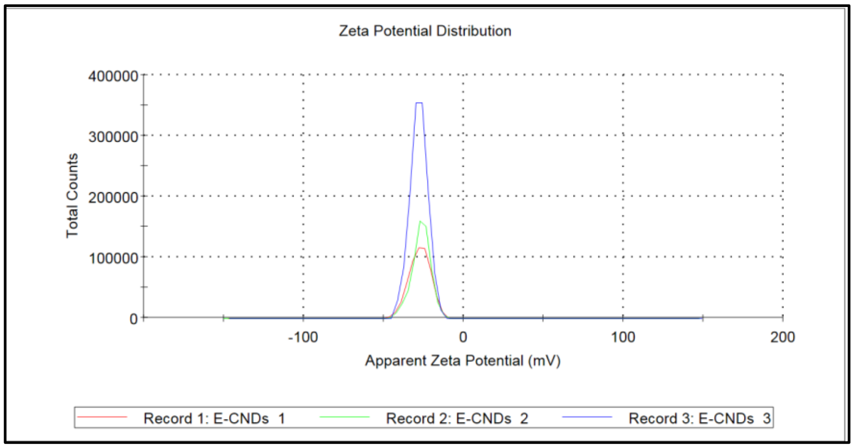
Zeta Potential Distribution
Total Counts
Apparent Zeta Potential (mV)
Record 1: E-CNDs 1
Record 2: E-CNDs 2
Record 3: E-CNDs 3

(a)

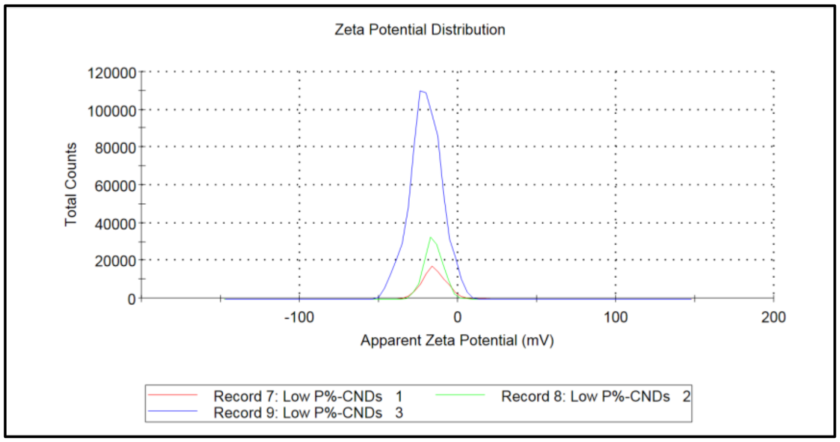
Zeta Potential Distribution
Total Counts
Apparent Zeta Potential (mV)
Record 7: Low P%-CNDs 1
Record 8: Low P%-CNDs 2
Record 9: Low P%-CNDs 3

(b)

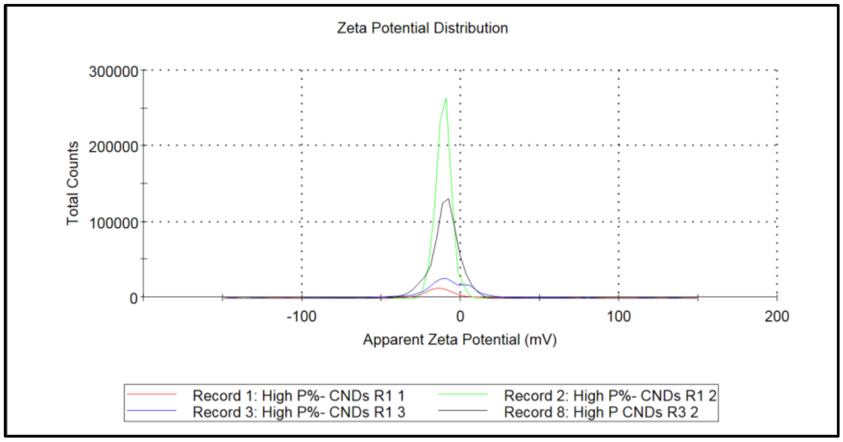
Zeta Potential Distribution
Total Counts
Apparent Zeta Potential (mV)
Record 1: High P%- CNDs R1 1
Record 2: High P%- CNDs R1 2
Record 3: High P%- CNDs R1 3
Record 8: High P CNDs R3 2

(c)

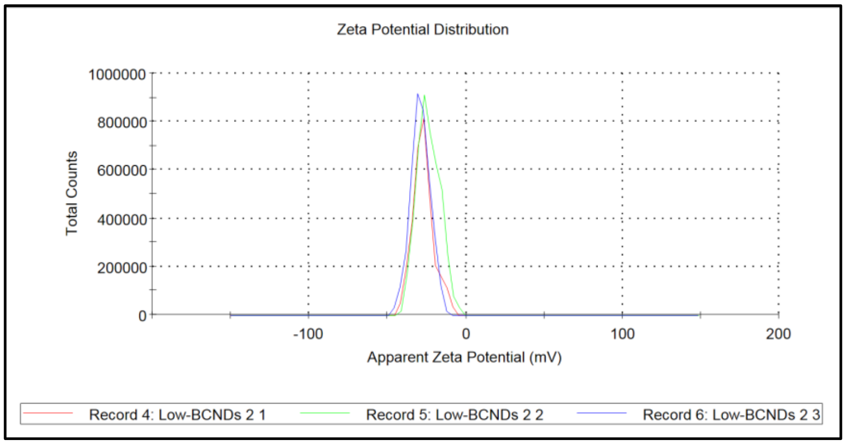

(d)

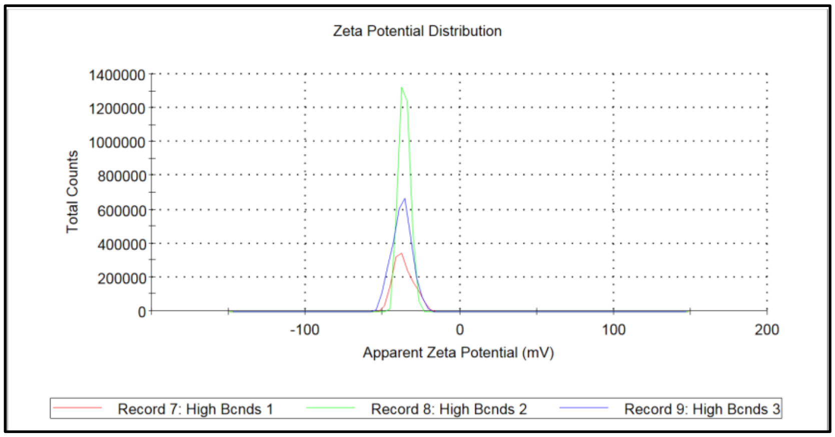

(e)

Figure S4. DLS graph of E-CNDs (a), Low P%-CNDs (b), High P%-CNDs (c), Low B%-CNDs(d), and High B%-CNDs (e).

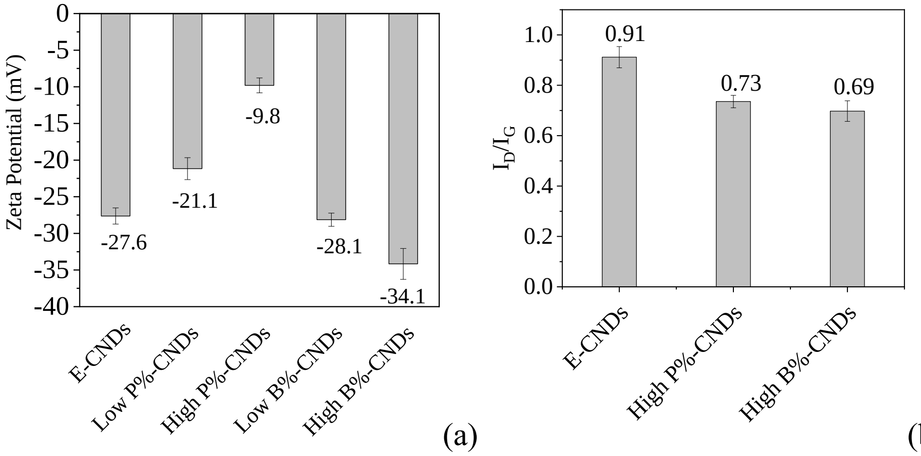

Figure S5. Zeta potential values of different CNDs samples (a), and ID/IG ratios of E-CNDs, High P%-CNDs, and High B%-CNDs (b).

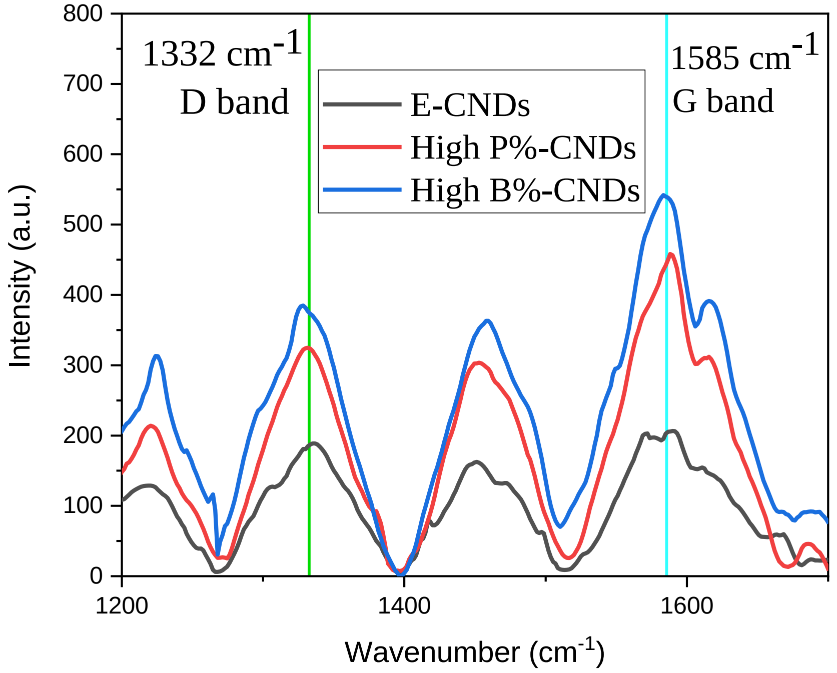

Figure S6. Raman spectra of E-CNDs, High P%-CNDs, and High B%-CNDs

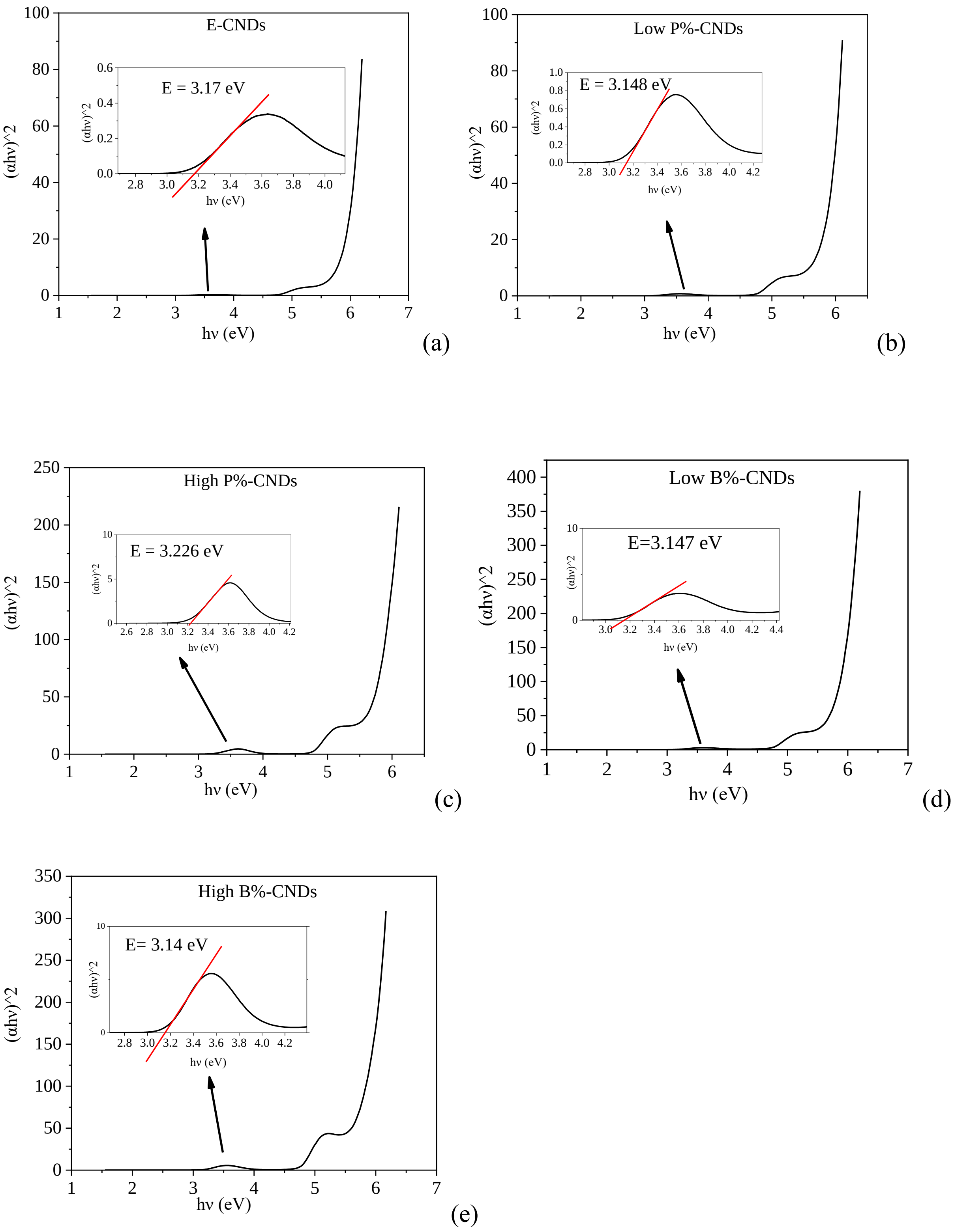

Figure S7. Tauc plots representing the band-gap energy of CND samples.

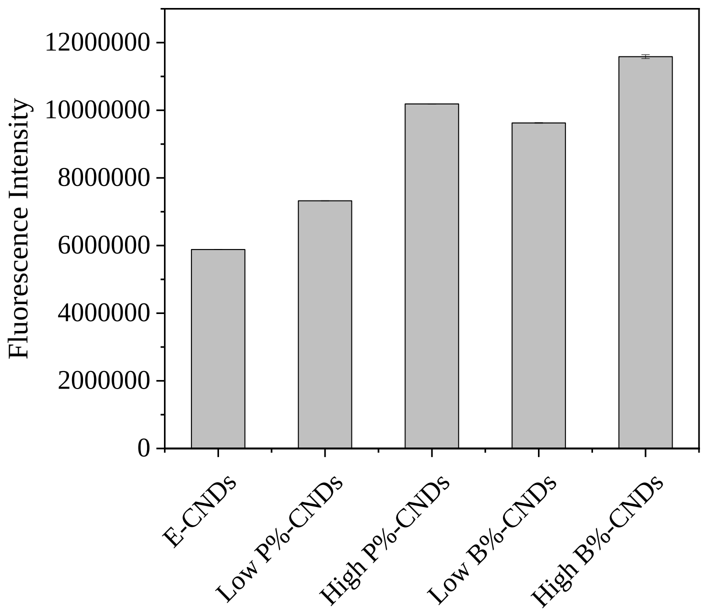

Figure S8. Fluorescence intensity of CND samples.

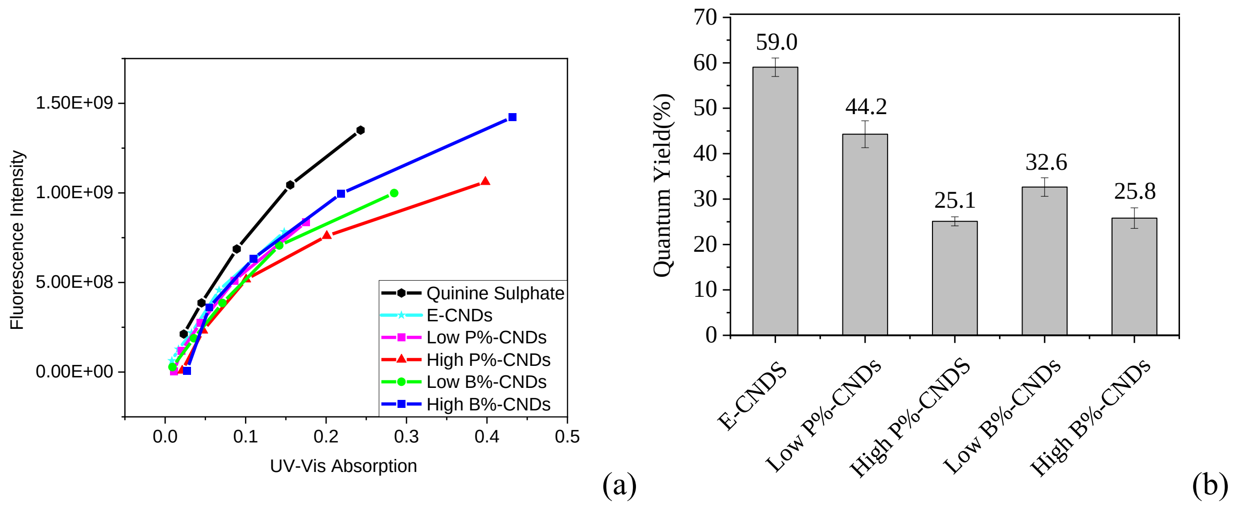

Figure S9. QY of CND samples compared to that of QS (a) and calculated QY of CND samples (b).

Table S1. Percentage of dopant elements across different CND samples extracted from XPS results.

CNDs Type	Dopant element atomic percentage	
	P	B
E-CNDs	0.04	0
Low P%-CNDs	0.38	0
High P%-CNDs	4.47	0
Low B%-CNDs	0	0.5
High B%-CNDs	0	1.95

Table S2. Atomic percentage of detected phosphorous chemical bonds by XPS.

	Low P%-CNDs	High P%-CNDs
P-C %	100	70.05
P-O %	0	29.09
P-N %	0	0.86

Table S3. Atomic percentage of detected boron chemical bonds by XPS.

	Low B%-CNDs	High B%-CNDs
B-COO %	87.04	0
B-O %	12.96	68.69
B-C %	0	27.68
B-N %	0	3.62

Table S4. I_D/I_G ratios and bond lengths in different CND samples. The bond length data were obtained from National Institute of Standards and Technology data base.[1]

CNDs type	E-CNDs	High P%-CNDs	High B%-CNDs	All CNDs
I_D/I_G ratio	0.91	0.73	0.69	–
Chemical Bond	C–N	C–P	C–B	C–C (average in graphite)
Length (pm)	117	156	149	142
Difference with C–C (%)	17.46	9.85	4.92	–

Table S5. Dominance and ratios of fit peaks' FWHM for E-CNDs, High P%-CNDs, and High B%-CNDs.

	E-CNDs	High P%-CNDs	High B%-CNDs
Dominance of Fit peak	1	1	2
FWHM of Fit peak 1/FWHM of Fit peak 2	0.63	0.62	0.58

References

(1) Computational Chemistry Comparison and Benchmark DataBase Standard - Reference Database 101, Experimental Diatomic bond lengths - Release 22 (May 2022) https://cccbdb.nist.gov/diatomicexpbondx.asp (Accessed 2023 -01 -21).
